
JOURNAL INFORMATION
==============================
NLM Title Abbreviation: Orthop Surg
Journal ID: Orthop Surg
NLM Journal ID: 101501666
Journal ID: OS

Orthopaedic Surgery

ISSN: 1757-7853
EISSN: 1757-7861
Publisher: Wiley

ARTICLE INFORMATION
==============================
PMCID: PMC11474715
DOI: 10.1111/os.13341
Article ID: OS13341
Article version: 1
Subjects: Issue Information, Issue Information

Issue Information

Electronic publication date: 2023 Jul 16
Publication date: 2023 Jul
Volume: 15
Issue: 7
First page: 1717
Last page: 1718
Copyright: © 2023 The Authors. Orthopaedic Surgery published by Tianjin Hospital and John Wiley & Sons Australia, Ltd.
License: This is an open access article under the terms of the http://creativecommons.org/licenses/by/4.0/ License, which permits use, distribution and reproduction in any medium, provided the original work is properly cited.
License URL: https://creativecommons.org/licenses/by/4.0/

Figure count: 0
Table count: 0
Page count: 5

source-schema-version-number: 2.0
cover-date: July 2023
details-of-publishers-convertor: Converter:WILEY_ML3GV2_TO_JATSPMC version:6.4.9 mode:remove_FC converted:15.10.2024

==============================

Volume 15 • Issue 7 • July 2023 • ISSN 1757-7861

### Orthopaedic Surgery

Open Access

REVIEW ARTICLES

1719
THE COMPARISON BETWEEN CEMENTED AND UNCEMENTED HEMIARTHROPLASTY IN PATIENTS WITH FEMORAL NECK FRACTURES: A SYSTEMATIC REVIEW AND META-ANALYSIS OF RANDOMIZED CONTROLLED TRIALS

1730
SYSTEMATIC REVIEW AND META-ANALYSIS OF LOCKED POSTERIOR DISLOCATION OF THE SHOULDER TREATED WITH SHOULDER ARTHROPLASTY: IMPROVED OUTCOMES FOR TOTAL SHOULDER ARTHROPLASTY ARE ASSOCIATED WITH INCREASED AGE

CLINICAL ARTICLES

1742
ASSESSMENT OF INSTANT LOSS OF ELBOW FLEXION IN CHILDREN WITH UNTREATED GARTLAND IIA HUMERAL SUPRACONDYLAR FRACTURES—A SIMULATION STUDY WITH LATERAL ELBOW RADIOGRAPHS

1749
PERCUTANEOUS FULL ENDOSCOPIC LUMBAR DISCECTOMY FOR SYMPTOMATIC ADJACENT SEGMENT DISEASE AFTER LUMBAR FUSION IN ELDERLY PATIENTS

1756
DOES PREOPERATIVE ACTIVITY LEVEL AFFECT POSTOPERATIVE CLINICAL OUTCOMES FOLLOWING HIP ARTHROSCOPY IN FEMOROACETABULAR IMPINGEMENT SYNDROME (FAIS) PATIENTS?

1763
FEASIBILITY ANALYSIS OF THE BONE CEMENT-GELATINE SPONGE COMPOSITE INTRAVERTEBRAL PREFILLING TECHNIQUE FOR REDUCING BONE CEMENT LEAKAGE IN STAGE I AND II KÜMMELL'S DISEASE: A PROSPECTIVE RANDOMIZED CONTROLLED TRIAL

1772
CLINICAL OUTCOMES OF CLOSED REDUCTION AND EXTERNAL FIXATION FOR ISOLATED SECOND METACARPAL BASE FRACTURE-DISLOCATIONS

1781
CLINICAL OUTCOMES OF OPEN-DOOR LAMINOPLASTY COMBINED WITH BILATERAL LATERAL MASS SCREW FIXATION FOR MULTI-LEVEL CERVICAL SPINAL STENOSIS WITH TRAUMATIC CERVICAL INSTABILITY AND SPINAL CORD INJURY: A RETROSPECTIVE STUDY

1790
THE RELIABILITY AND ACCURACY OF THE MEDIAL MALLEOLAR FRACTURE CLASSIFICATION BASED ON 3D CT RECONSTRUCTION

1799
THE INFLUENCE OF SAGITTAL ANGLE OF POSTERIOR MALLEOLUS FRACTURE ON ANKLE JOINT STABILITY: A RETROSPECTIVE STUDY OF 120 CASES

1806
PREOPERATIVE PREVALENCE AND RISK FACTORS FOR CALF MUSCULAR VEIN THROMBOSIS IN ELDERLY PATIENTS WITH HIP FRACTURE

1814
INCIDENCE OF AND RISK FACTORS FOR SCREW LOOSENING AFTER ILIOSACRAL SCREW FIXATION FOR POSTERIOR PELVIC RING INJURY

1823
OPERATIVE EFFECT COMPARISON OF FLEXIBLE DRILL GUIDING *VS.* TRADITIONAL DRILL GUIDING TEMPLATE FOR LOWER CERVICAL PEDICLE SCREW INSERTION: A RETROSPECTIVE ANALYSIS

1831
TREATMENT OF EARLY UNDIFFERENTIATED CHRONIC MONOARTHRITIS OF THE WRIST BY ARTHROSCOPIC WRIST SYNOVECTOMY COMBINED WITH PARTIAL DENERVATION

1839
COMPARISON BETWEEN ULTRASOUND-GUIDED PERICAPSULAR NERVE GROUP BLOCK AND LOCAL INFILTRATION ANALGESIA FOR POSTOPERATIVE ANALGESIA AFTER TOTAL HIP ARTHROPLASTY: A PROSPECTIVE RANDOMIZED CONTROLLED TRIAL

1847
VALGUS STRESS RADIOGRAPH CAN PREDICT ALIGNMENT CHANGE OF MEDIAL MOBILE-BEARING UNICOMPARTMENTAL KNEE ARTHROPLASTY

1854
QUALITY OF LIFE AND DEPRESSION STATUS OF CAREGIVERS OF PATIENTS WITH FEMORAL NECK OR INTERTROCHANTERIC FEMORAL FRACTURES DURING THE FIRST YEAR AFTER FRACTURE TREATMENT

RESEARCH ARTICLES

1862
COMPARISON OF THE FEMORAL CONDYLAR ELLIPSE LINE AND THE SURGICAL EPICONDYLAR AXIS: 3D MEASUREMENT BY MRI SCANS IN HEALTHY CHINESE PEOPLE

1870
A MULTI-FACTOR ANALYSIS OF REVISION IN TOTAL HIP REPLACEMENT USING THE COLLARLESS-POLISHED-TAPERED STEMS WITH DIFFERENT CUPS

1884
COMPENSATION CHARACTERISTICS OF SPINAL SAGITTAL MALALIGNMENT ACCORDING TO PELVIC INCIDENCE IN ELDERLY PATIENTS WITH DEGENERATIVE LUMBAR SPINAL STENOSIS

OPERATIVE TECHNIQUE

1893
TECHNICAL NOTES AND CLINICAL OUTCOMES OF FULL-ENDOSCOPIC INTERBODY FUSION VIA TRANSFORAMINAL APPROACH FOR HARD DISC HERNIATIONS IN THORACOLUMBAR JUNCTION

CASE REPORTS

1904
FROM THE DESTRUCTION OF TWO LUMBAR SEGMENTS TO THORACIC-LUMBAR-PELVIC FUSION: A CASE CAUSED BY CONGENITAL INSENSITIVITY TO PAIN WITH ANHIDROSIS AND LITERATURE REVIEW

1915
THE LONGEST KNOWN FOLLOW-UP OF VITALLIUM MOLD ARTHROPLASTY IN CHINA: A CASE REPORT AND LITERATURE REVIEW

1920
A NOVEL SURGICAL TECHNIQUE FOR NEGLECTED VOLAR DISLOCATION OF THE LUNATE: CASES REPORT AND LITERATURE REVIEW

WILEY

View this journal online at wileyonlinelibrary.com/journal/os

### Orthopaedic Surgery

**Editor-in-Chief**
Gui-xing Qiu, *Beijing, China*
Francis John Hornicek, *Boston, USA*

**Associate Editor**
Yong-cheng Hu, *Tianjin, China*
Thomas Blattert, *Leipzig, Germany*
Douglas Letson, *Tampa, USA*
Xin-long Ma, *Tianjin, China*
Ling Qin, *Hong Kong, China*

**Editorial Board**
Giovanni Alessi, *Gent, Belgium*
Haluk Berk, *Izmir, Turkey*
Thomas Blattert, *Leipzig, Germany*
Mathis Bostrom, *New York, USA*
Kao-wha Chang, *Taiwan, China*
Edmund YS Chao, *Baltimore, USA*
Liao-bin Chen, *Wuhan, China*
Yan-xi Chen, *Shanghai, China*
Kenneth MC Cheung, *Hong Kong, China*
Thomas L. Clemens, *Birmingham, USA*
Benny Thellefsen Dahl, *Copenhagen, Denmark*
Lian-fu Deng, *Shanghai, China*
Jean Destandau, *Bordeaux, France*
Jian Dong, *Shanghai, China*
Shun-wu Fan, *Hangzhou, China*
David Fang, *Hong Kong, China*
Shi-jun Gao, *Shijiazhuang, China*
Wei Guo, *Beijing, China*
Yong Hai, *Beijing, China*
Alan R. Hargens, *San Diego, USA*
Francis John Hornicek, *Boston, USA*
William C. Horton, *Atlanta, USA*
John Hsiang, *Seattle, USA*
Yong-cheng Hu, *Tianjin, China*
Qing Jiang, *Nanjing, China*
Paul Jutte, *Groningen, Netherlands*
Jean Pierre Kalala, *Gent, Belgium*
Choon-Ki Lee, *Seoul, Korea*
Jean Jacque Lehuec, *Bordeaux, France*
Lawrence G. Lenke, *Seattle, USA*
Douglas Letson, *Tampa, USA*
Kwok Sui Leung, *Hong Kong, China*
Jian-hao Lin, *Beijing, China*
Bao-ge Liu, *Beijing, China*
Wei-hee Lo, *Taiwan, China*
William Weijia Lu, *Hong Kong, China*
Keith DK Luk, *Hong Kong, China*
Zong-ping Luo, *Suzhou, China*
Cyril Mauffrey, *Denver, USA*
Wei-hu Ma, *Ningbo, China*
Xin-long Ma, *Tianjin, China*
Ling Qin, *Hong Kong, China*
Gui-xing Qiu, *Beijing, China*
Axel Reinhardt, *Potsdam, Germany*
Pu-yi Sheng, *Guangzhou, China*
Harry L Shufflebarger, *Miami, USA*
David Stulberg, *Chicago, USA*
Paul Sung, *San Diego, USA*
Paul Unwin, *Leicester, UK*
Luc F. De Waele, *Gent, Belgium*
Fei Wang, *Shijiazhuang, China*
Wei-jun Wang, *Nanjing, China*
James N. Weinstein, *Chicago, USA*
Hee-Kit Wong, *Singapore*
Su-jia Wu, *Nanjing, China*
Jian-ru Xiao, *Shanghai, China*
Hui-lin Yang, *Suzhou, China*
Tzai-chiu Yu, *Taiwan, China*
Xi-jie Yu, *Chengdu, China*
Hansen A. Yuan, *New York, USA*
Hong-wen Zhu, *New York, USA*

**Address for Editorial Correspondence:** Editorial Board of *Orthopaedic Surgery*, Tianjin Hospital, 122 Munan Road, Tianjin 300050, China (email: orthopaedicsurgery@126.com; telephone: +86 22 2833 4734; fax: +86 22 2824 1184).

**Disclaimer:** The Publisher, Tianjin Hospital and Editors cannot be held responsible for errors or any consequences arising from the use of information contained in this journal; the views and opinions expressed do not necessarily reflect those of the Publisher, Tianjin Hospital and Editors, neither does the publication of advertisements constitute any endorsement by the Publisher, Tianjin Hospital and Editors of the products advertised.

**Copyright © 2023 The Authors. Orthopaedic Surgery published by Tianjin Hospital and John Wiley & Sons Australia, Ltd**

**Copyright and Copying (in any format)**
Copyright © 2023 The Authors. Orthopaedic Surgery published by Tianjin Hospital and John Wiley & Sons Australia, Ltd. All rights reserved. No part of this publication may be reproduced, stored or transmitted in any form or by any means without the prior permission in writing from the copyright holder. Authorization to copy items for internal and personal use is granted by the copyright holder for libraries and other users registered with their local Reproduction Rights Organisation (RRO), e.g. Copyright Clearance Center (CCC), 222 Rosewood Drive, Danvers, MA 01923, USA (www.copyright.com), provided the appropriate fee is paid directly to the RRO. This consent does not extend to other kinds of copying such as copying for general distribution, for advertising or promotional purposes, for republication, for creating new collective works or for resale. Permissions for such reuse can be obtained using the RightsLink "Request Permissions" link on Wiley Online Library. Special requests should be addressed to: permissions@wiley.com

For submission instructions, subscription and all other information visit: http://wileyonlinelibrary.com/journal/os

ISSN 1757-7861 (Online)

OS.JEB.July23
